# Cesar Fernandez-de-las-Penas, Leon Chaitow, and Jean Schoenen (eds.): Multidisciplinary management of migraine: Jones & Bartlett Learning/Publication date: 2/5/2012

**DOI:** 10.1007/s10194-012-0488-8

**Published:** 2012-10-14

**Authors:** Aksel Siva

**Affiliations:** Cerrahpaşa School of Medicine, Istanbul University, Istanbul, Turkey

A book to read: *Multidisciplinary management of migraine*

In this era of e-books and e-reading, as an old fashioned individual I love reading paper-books, sitting in my armchair and hold them in my hands, go through pages… And, I love more to read good books. This is one of them. It covers all aspects of migraine and even more. As in the preface stated by Professor Olesen, the editorial trio is a combination of a physical therapy physician, an osteopath and finally a neurologist, a well-known and moderate headache expert, Prof Schoenen. This unique editorialship is also reflected in the combination of the contributing authors and the chapters of the book.

The first part of this book covers what a classical migraine textbook would cover—basic and clinical information on different aspects of migraine and then goes on chapters on its management written mostly by non-neurologist reflecting their understanding and management of migraine and headaches. The reader will find a large number of reviews on complementary and alternative therapeutic options currently used in migraine, which the editors outline as alternative medical systems, biologically based therapies, manual therapies and mind–body therapies.

I should confess that, as a relatively conservative neurologist, I had some doubts when I first took the book in my hands. However, when I started to read through different chapters, I became more broad-minded and less critical! The non-neuro-disciplinary approach in some of the chapters and various “other” management issues expand the alternatives of migraine treatment. The wide spectrum of additional non-pharmacological treatments covered in the book provides the almost unlimited options of what can be done with a migraine/headache patient. This do not mean that one would agree and consider to include all these forms of management in their practice, which involve a wide variety of practices from the traditional to the most modern forms of management, from the emerging invasive techniques to the manipulative approaches, from the behavioral to the pharmacological ones or any of their combinations.

A few typo errors such “agonists” Instead of “antagonists” may be inevitable….

In summary, the chapters are easy to read, cover all the necessary and updated information a print-book can allow. If you look for a high-science book, this is not going to be among the top listed ones, but who needs another one anyway? It is a book for the practicing headache physician and expands our thinking on migraine and on the different forms of available pharmacological and non-pharmacological therapies. So, for the ones who share my understanding, “Multidisciplinary Management of Migraine” is a different book to read and think.

